# Technology as Teammate: Examining the Role of External Cognition in Support of Team Cognitive Processes

**DOI:** 10.3389/fpsyg.2016.01531

**Published:** 2016-10-07

**Authors:** Stephen M. Fiore, Travis J. Wiltshire

**Affiliations:** ^1^Department of Philosophy and Institute for Simulation & Training, University of Central Florida, OrlandoFL, USA; ^2^Dynamical Systems Lab, Department of Psychology, University of Utah, Salt Lake CityUT, USA

**Keywords:** team cognition, macrocognition in teams, external team cognition, teamwork, taskwork, oﬄoading, scaffolding

## Abstract

In this paper we advance team theory by describing how cognition occurs across the distribution of members and the artifacts and technology that support their efforts. We draw from complementary theorizing coming out of cognitive engineering and cognitive science that views forms of cognition as external and extended and integrate this with theorizing on macrocognition in teams. Two frameworks are described that provide the groundwork for advancing theory and aid in the development of more precise measures for understanding team cognition via focus on artifacts and the technologies supporting their development and use. This includes distinctions between teamwork and taskwork and the notion of general and specific competencies from the organizational sciences along with the concepts of oﬄoading and scaffolding from the cognitive sciences. This paper contributes to the team cognition literature along multiple lines. First, it aids theory development by synthesizing a broad set of perspectives on the varied forms of cognition emerging in complex collaborative contexts. Second, it supports research by providing diagnostic guidelines to study how artifacts are related to team cognition. Finally, it supports information systems designers by more precisely describing how to conceptualize team-supporting technology and artifacts. As such, it provides a means to more richly understand process and performance as it occurs within sociotechnical systems. Our overarching objective is to show how team cognition can both be more clearly conceptualized and more precisely measured by integrating theory from cognitive engineering and the cognitive and organizational sciences.

## Introduction

Organizations are often characterized as complex sociotechnical systems that require effective coordinative and collaborative cognitive processes across individuals and teams in order to meet their goals. As such, research in team cognition has become increasingly prevalent over the past decade. Team cognition is a broad area of research meant to explore the manifestation of cognition in the context of teamwork (Salas and Fiore, 2004; Letsky et al., 2008; Salas et al., 2012; Turner et al., 2014). This includes understanding how memory influences teams (e.g., transactive memory systems, Lewis and Herndon, 2011), or how cognitive constructs, such as mental models, can provide explanatory value to inform team functioning (e.g., shared mental models, DeChurch and Mesmer-Magnus, 2010b). Other processes such as attention and decision making, as they arise in teams, have also been studied [e.g., distributed cognition, Hutchins, 1995a; distributed situation awareness (DSA), Stanton, 2016]. A significant amount of research on these topics has been able to inform our understanding of teams and how, for example, training (Cooke and Fiore, 2009) or system design (Kiekel and Cooke, 2004; Bowers et al., 2006) can be improved.

We suggest, however, that what constitutes cognition in the organizational sciences is too often narrowly construed. This potentially leads to an incomplete understanding of team processes and the many factors leading to successful performance, particularly when teams are made up of a hybrid of humans and technology. Specifically, despite a large body of research, there is less attention paid to external cognition, that is, artifacts or material objects used in service of team cognition, or technologies supporting their development and use, and how these relate to team effectiveness. In the more general study of teams, there have been discussions of teams and their relation to technology. For example, when viewing teams as a human-technology system (Kozlowski et al., 2015), researchers describe how the technological sub-system is an important component to understanding the kinds of emergent processes typically related to team effectiveness (e.g., cohesion or collective efficacy). Others have noted how the technology, itself, can shape communicative and coordinative interactions and, thus, substantially influence team process (Bell and Kozlowski, 2012). Nonetheless, studies of technology, and the artifacts it helps teams produce, is under represented in the team cognition literature.

As evidence of this lack of inquiry within the field of team cognition, recent reviews have not made mention of artifacts or associated terms, or even of technology, in any substantial way. For example, although drawing from multiple disciplines and providing what is described as a “cross-domain review” on the measurement of team cognition, there was no mention of how external cognition factors like material objects, artifacts, or technology should be considered as part of the team process (Wildman et al., 2014). In a review on the role of team knowledge in understanding collaborative processes, despite a comprehensive coverage of the ways knowledge is conceptualized, there is no mention of how these external cognition factors artifacts relate to knowledge construction and use, nor how they should fit within team cognition research (Wildman et al., 2012). Similarly, in a meta-analysis of team cognition constructs, these factors were not considered in any of the classifications that examined the relationship between team cognition and performance outcomes (Turner et al., 2014).

What is striking about these and earlier similar articles (e.g., DeChurch and Mesmer-Magnus, 2010a,b), is that many of the studies making up the foundation for these reviews, in some form or another, used technologies that would create or need artifacts for task completion. As an example, this could include project management type tasks where planning required the creation or use of artifacts, or computer-based experimental tasks (e.g., simulations of aviation necessitating use of diagrams), to even just technologies supporting information sharing and storage (e.g., chat boards). Our point is that there is tremendous potential in considering these externalized cognition factors as a relevant element of team cognition. From this, research can examine the degree to which it may moderate or mediate any number of team process and performance outcomes and help us understand and improve team cognition.

In short, we suggest that team cognition research lacks the conceptual scaffolds necessary to examine how artifacts and associated technologies are related to team process and performance. To redress this gap, we integrate a set of constructs under the general label of external cognition to describe how the concept of artifacts, and the technology supporting their development and use, have been discussed as a foundational part of collaboration across a number of fields. With that as a stepping off point, we then show how distinctions between teamwork and taskwork, arising from organizational theory on team training, and differences between oﬄoading and scaffolding cognition, arising from the cognitive sciences, can be united to provide a framework that advances team cognition research. Our goal is to show how these provide explanatory value to team cognition theory by helping to conceptualize technology as teammate.

This paper consists of two major sections, each with two subsections. First we provide an overview of the general idea of technology in team cognition in the context of research and theory on complex collaborative environments where technology is inherent and cognition is often externalized. Second, using the general label of “artifact” we describe how external forms of cognition have been examined in a variety of settings so as to provide evidence for the reach of this idea and how it has been related to cognition and collaboration. This initial half of the paper provides the foundational literature on which we build the argument for examining technology, broadly construed, as part of a team. The latter half works to integrate ideas from organizational research on teams, and concepts from cognitive science, to provide a novel means through which to understand team cognition. Specifically, in the third section, we discuss the distinction between “teamwork” and “taskwork” – ideas that have yet to be integrated with the external cognition perspective. Fourth, we bring in ideas from cognitive science about oﬄoading and scaffolding cognition to show how these help us more finely distinguish between forms of external cognition in the context of teams. Within these sections we provide guidelines and research questions devised around technology in support of external cognition to help researchers examine teams as socio-technical systems.

## Cognition, Technology, and Teams

In an age of ubiquitous technology, the study of team cognition needs research that more closely examines our assumptions about what is cognition and its manifestation through, and within, technology in the modern workplace. This is necessary to develop the next phase of team cognition research for the organizational sciences. Indeed, there have been recent calls for research on teams to improve understanding human-system issues arising from the team-technology integration. For example, Bell and Kozlowski (2012) called out the lack of studies in organizational research that have fully examined the complementarity between technology and team interaction and how they lead to emergent states. In this context, they specifically labeled such issues as one of the important themes for future research on teams. More recently, Kozlowski et al. (2015) noted the criticality of understanding how workflow within teams, interacts with technology to influence cognition and behavior. They highlight the need for more research on team design and, included in this, is a need for research that examines how technologies can help or hinder numerous factors related to team cognitive factors (e.g., information sharing and distribution).

Toward this end, drawing from research focusing on the intersection of cognition and technology as it occurs in naturalistic and dynamic organizational contexts (Cacciabue and Hollnagel, 1995; Pennathur et al., 2008; Jenkins et al., 2011; Fiore, 2012; Cooke et al., 2013; Lee and Kirlik, 2013; Gorman, 2014), we integrate theory from cognitive engineering with the cognitive and organizational sciences in order to help team researchers more fully conceptualize cognition in its varied forms. We show how the next phase of team cognition research can be pursued as a form of team-technology hybrid wherein we can come to better understand the tight coupling between the individual, the team, and the technologies they rely upon.

Our main argument is that understanding team cognition as it occurs in real-world work settings requires an expanded view where cognition is seen as distributed and context dependent in a social environment in which artifacts often support cognitive functions (Suchman, 1987, 2007; Hutchins, 1995a; Clancey, 1997; Hollnagel, 2002). Specifically, we advance the notion that artifacts support cognition by enabling the transition and development of internalized knowledge held by team members to externalized knowledge held at the team-level (Fiore et al., 2010b; Rentsch et al., 2010, 2014). We draw from a diverse body of research and theory to emphasize that the functions of cognition can, and must be, viewed as sometimes occurring, not just “in” the head, but also “outside the head”; that is, viewing cognition in a broader context as distributed across the boundaries of brains, bodies, and environment (Fiore, 2012; Cooke et al., 2013; Gorman, 2014). We describe DSA theory (e.g., Stanton, 2016), interactive team cognition (ITC) theory (e.g., Cooke and Gorman, 2009; Cooke et al., 2013), and macrocognition in teams (MiTs) theory (Fiore et al., 2008, 2010b,c) from cognitive engineering, and extended cognition theory from cognitive science (Clark and Chalmers, 1998; Clark, 2001a,b), to better understand the increasingly prevalent role technology plays as a form of external cognition in complex collaborative work domains.

The combination of these perspectives provides a strong foundation from which the organizational sciences can begin to consider and measure external team cognition in order to contribute to team theory and practice and, in turn, increase organizational effectiveness. We now turn to a discussion of theory that has broadly considered how contextual factors, like technology, play a role in team process.

### Considerations of Context and Team Cognition

The 20th century saw tremendous gains in organizational productivity thanks to numerous technological advances. As mechanization began to dominate in the early decades, work practices changed and humans adapted to these new systems. Importantly, organizational scientists studying these changes recognized that not all adaptations were equal. In the middle part of the century, researchers with the Tavistock Institute observed innovative work practices that moved beyond bureaucratization and mechanization to create a new form of work. In the British mining industry, where technology had made tremendous inroads, some workers had developed a higher form of collaboration between themselves and their technology (for a discussion, see Trist, 1981). Viewed as a sub-system of the organization within which autonomy had been enhanced, it could lead to greater group cohesion, self-regulation and coordination as teams developed new practices for working with each other and the new technologies. This was seen as an important alternative to Tayloresque and Weberian approaches in that, for organizational design, “the best match would be sought between the requirements of the social and technical systems” (p. 9). In many respects, this revolutionized organizational theory by introducing systems thinking into the lexicon and helping to produce a more holistic view of the interactions between, people, machines, and the environmental context in which they operate (Trist, 1981).

We open this section with this brief historical perspective because, although socio-technical systems theory was an important part of organizational research, and originated from a study of groups working with technology, this perspective had less influence on the study of teams. Research in teams throughout most of the 20th century focused more on the social than the technical (e.g., Guzzo and Dickson, 1996). Furthermore, with the advent of the cognitive revolution in the organizational sciences, we saw an infusion of research on the interaction of the social and the cognitive (Hinsz et al., 1988, 1997; Lord and Maher, 1991; Larson and Christensen, 1993), but, still, with little incorporation of technology’s role in teams. Rather, this led to the emergence of the study of team cognition and the manifestation of cognition within and across individuals during complex and dynamic interactions (e.g., Salas and Fiore, 2004).

From this we gained significant understanding of how social and cognitive factors influence process and performance. For example, a tremendous amount of research has studied the relationship between team knowledge, such as shared mental models, and team outcomes (DeChurch and Mesmer-Magnus, 2010b). Research has also studied how coordination is altered by expertise within the team (e.g., Faraj and Sproull, 2000; Espinosa et al., 2004, 2007), or how coordinative mechanisms are necessary for reaching shared goals or achieving desired performance outcomes (Gittell and Weiss, 2004; Gittell, 2006; Brodbeck et al., 2007; Okhuysen and Bechky, 2009). In brief, there has been a pervasive emphasis on the role of stable mental constructs such as shared knowledge and/or coordination processes. But these cognitive structures are still abstract, subjective, internal, and provide a restricting view of cognition to the organizational sciences (e.g., Mitchell et al., 2011). This research now transcends disciplines and many theories, methods, and domains are part of team cognition research (Salas et al., 2012).

Despite these theoretical and empirical advances in numerous areas, most organizational research on teams has not taken into account how the environment in general, and technology, in particular, interacts with individual and team cognition. Recent efforts have called for stronger integration of these approaches (e.g., Rico et al., 2011) as well as for a broader perspective on what is meant by team cognition and how interaction dynamics and context are related to team effectiveness (Fiore et al., 2010a; Cooke et al., 2013; Cooke, 2015). Along these lines, we argue that team research in the organizational sciences will benefit from theories emerging in other disciplines that more fully account for the role of contextual factors in team cognition in general, and the role of technology, in particular. Generally, these theories consider cognition as something more than that which goes on “inside the head”; rather, cognition is something that can be studied both *inside and outside the head* as team members interact with each other and their technology. We next briefly review some of this theorizing.

#### Context and Behavior When Interacting with Technology

In early theorizing in this area, research on *situated cognition*, by social anthropologist, Suchman (1987), argued that the agent and the environment have to be included in theorizing about cognition. Her research emphasized the role of context in cognition and the use of ethnomethodology to analyze human activity arising between a person and the setting in which that activity takes place. From this, researchers began to recognize relational coupling between situation and action, where meaning is constructed within particular contexts (Fiore, 2013).

Even information processing theorists made a claim for the value of understanding cognition as situated (Vera and Simon, 1993). They argued that symbolic and representational approaches could explain interactions with complex work systems. From this perspective, simulation of cognitive activity can be conceived of as occurring within and across individuals and the representational systems on which they rely (see also Larkin and Simon, 1987).

Coming out of research on cognitive engineering, DSA theory was another to examine context to place emphasis on understanding the social-technical system in its entirety as the unit of analysis (Stanton et al., 2006; Stanton, 2016). While DSA proposes that researchers delineate between their adopted unit of analysis such as ‘in mind,’ ‘in world,’ or ‘in-interaction,’ the focus of DSA is typically on the behavioral interactions that facilitate the transaction of awareness amongst agents in a socio-technical system, whether those are social sub-systems (e.g., individual humans and teams) or technical sub-systems (e.g., technologies, interfaces, artifacts, displays, etc.). In this case, situation awareness refers to holding information regarding the status of a given situation. But DSA differs from traditional notions of SA (Endsley, 1995) in that is does not assume that SA can be held only “in-mind” of humans, but rather it can be distributed across the technologies as well and is available to the human as needed.

When considering SA in teams, empirical work comparing a DSA approach to a traditional team cognition approach on shared SA, found that teams who had awareness that was more differentially distributed across team members (as shown by concept maps) performed better than teams who shared more information and held largely the same awareness on a rogue vehicle detection task (Kitchin and Baber, 2016). In another example, teams working in anesthesia management were shown to explicitly rely on their interactions with artifacts such as computer monitors and whiteboards, as well as their teammates to gain the appropriate awareness that allowed them to perform their duties effectively (Fioratou et al., 2016). Other studies have similarly shown that it is more important that awareness is distributed across team members and their technologies and that such cases often exhibit improved task performance (e.g., Bourbousson et al., 2011; Sorensen and Stanton, 2013).

Coming out of the cognitive sciences, others have similarly conceptualized and examined team cognition and behaviors at the collective level. Specifically, ITC theory (Cooke and Gorman, 2009; Cooke et al., 2013; Cooke, 2015) draws from post-information processing perspectives of individual cognition, such as embodied cognition and activity theory. ITC views team cognition more dynamically, as an activity engaged by teams over time and, in line with earlier views of situated cognition (e.g., Suchman, 1987), sees cognition as inseparable from context. Similar to DSA, an important tenant of ITC is that team cognition needs to be examined at the level of the team (e.g., communication; Cooke et al., 2008, 2004). Finally, it differs primarily from traditional theories of team cognition by arguing that performance differences can be more accurately understood, not by knowledge differences in the team (e.g., shared mental models), but in the behavioral interactions (Cooke et al., 2009; Gorman et al., 2010).

Empirical evidence for ITC theory comes from findings where the disruption of interactions patterns during task training actually improve later performance when compared to those whose interaction patterns were not disrupted (Gorman et al., 2010). Teams that were disrupted learned to adapt interaction behaviors that later proved beneficial. Other results show that, while team performance increases across a full series of performance events, changes to team knowledge occur primarily during earlier events, whereas, changes and refinements to the team’s interactive processes occurs during more of the missions (Cooke et al., 2001). This suggests that the collective and interactive behaviors are what is driving the continued team performance improvements, rather than the continued development of task knowledge.

In sum, the argument that theorizing on collaborative cognition should account for contextual and technological factors, has been an important part of research on teams operating in complex settings. These views converge on the perspective that cognition can occur at the intersection of the individual, the team, their technology, and the environment, to influence their behaviors in context. This work makes strides in helping us see how features and components of tasks can be distributed across team member’s internal cognitive systems, the collective external cognitive system of the team, as well as across artifacts and technologies in the environments in which they interact (Zhang and Norman, 1994; Zhang, 1998; Hutchins, 1999; Stanton et al., 2006; Clark, 2008; Fiore et al., 2010b; Cooke et al., 2013).

We build from this to argue that external cognition as part of that context, whether it be physical, mechanical, technological or otherwise, needs to be recognized and measured as a part of team cognition. This, then, can be used to help us understand and measure *where* the team is being supported by these as well as *how*. In this way, we add to team cognition research by focusing on the ways in which teams collaborate with each other *and* with/through technology. We next discuss how MiTs theory, an approach aligned with these perspectives, can advance research on teams. We focus on the role of technology in support of external cognition to provide theoretical guidance that can facilitate empirical work in this area.

#### External Cognition and Macrocognition in Teams

Researchers studying cognition embedded in rich, real-world environments, developed the concept of *macrocognition*, a term that embodies a shift away from the traditional micro-view of cognition to describe how cognition operates when faced with complexity (Hollnagel, 2002). Broadly, macrocognition includes the ideas that: (a) across natural and artificial cognitive systems, the process and product of cognition will be distributed; (b) cognition is not self-contained and finite, but a continuance of activity; (c) cognition is contextually embedded within a social environment; (d) cognitive activity is not stagnant, but dynamic; and (e) artifacts aid in nearly every cognitive action (Hollnagel, 2002; Klein et al., 2003, 2006; Fiore, 2012). These ideas provide important additional explanatory power by providing an enhanced appreciation of how interaction unfolds in dynamic and contextually rich settings.

Macrocognition in teams theory is an interdisciplinary integration of much of this prior research on collaborative cognition that emphasizes both internalized and externalized cognition and the role of artifacts in collaboration (Fiore et al., 2008, 2010b,c). In addition to considering how, for example, shared memory structures support teamwork (e.g., understanding how team mental models help sequence actions), MiTs theory focuses on ways in which internalized knowledge is transformed to externalized knowledge by both individual and team-level cognitive processes for the purposes of knowledge coordination (Fiore et al., 2010b). In this way, it addresses how teams externalize cognition to collaboratively build knowledge through the transformation of data to information to knowledge in service of team problem solving (Fiore et al., 2010b). The macrocognitive view is particularly relevant to this paper given that prior theorizing specifically emphasized how individuals and teams deal with complexity via reliance on technology (e.g., Hollnagel, 2002; Klein et al., 2006).

Foundational to MiT theory is the notion of *extended cognition* (Clark and Chalmers, 1998; Clark, 2001a). Similar to the theorizing discussed earlier, this perspective argues that the brain is inextricably coupled to one’s external environment and often relies on this coupling for many complex tasks. The extended cognition perspective also posits that some of what is normally construed as cognition localized “within the head,” can also occur beyond the boundaries of the head, that is, as externalized cognition. Two simple examples of extended cognition include note-taking during a lecture and working out a mathematical problem on paper. Broadly, the former is an act of “remembering” in the sense that this is a type of external storage to which one can later refer. The latter is an act of “cognition” in the sense that the mental effort required to solve the problem is off-loaded onto the environment (i.e., calculations are not all done entirely mentally).

More generally, if a given task requires the temporarily formed and synergistic coalition of the body’s sensorimotor systems and neural circuits, as well as artifacts and/or other people in the environment, then it is difficult to relegate the functions of cognition to just occurring within the head (Anderson et al., 2012). Note that the extended view of cognition does not claim that the brain is not playing a crucial role in cognition. Rather, the point is that the role of the brain, at least in this respect, is to act “as a mediating factor in a variety of complex and iterated processes which continually loop between brain, body, and technological environment” (Clark, 2002, p. 24). Through this theoretical lens, cognitive functions can be construed of as extending outside of the body, that is, externalized. Of course, to include artifacts as part of cognition is contingent upon the notion that their use must be available when needed and accessed in ways analogous to traditional retrieval mechanisms (Clark, 2001b). This sociotechnical system is the foundation from which solutions to complex problems can emerge (Fiore et al., 2008).

In their theorizing on MiTs, Fiore et al. (2010b,c) wove this into an elaboration of the functional role externalized cognition plays in collaborative problem solving. Motivation for this claim stems from the notion that “the degree the team-task requires the construction of a shared understanding, external representational tools can act as a scaffolding to facilitate the building of that shared representation” (Fiore and Schooler, 2004, p. 134). Building on this, in MiT theory, externalized cognition can be a focal point for team discussion and elaboration, and can support analysis of ideas put forth, and potential solutions, by helping members attend to key details articulated in the externalization. In this view, externalized cognition is particularly useful when teams are supported by technology; that is, by sociotechnical systems devised to help members deal with the tremendous variety of data and information with which they are confronted when dealing with complex problems (cf. Klein et al., 2003, 2006).

A key gap in the theorizing on MiT theory, though, is that it does not fully articulate the richness of what is meant by externalized cognition. Although it describes external cognition as an important component of knowledge building in teams, the specific ways in which external cognition can manifest itself, and how it plays a role in extending team cognition, need to be better articulated. We next address this gap in MiT theory via explication of artifacts as externalized cognition and articulation of the specific ways these play a role in different aspects of team cognition. Toward this end, we summarize some of the prior research on which the MiT theory was built and which specifically focuses on the idea of externalized cognition and role artifacts play when collaborating.

### Artifacts and Technological Support as Externalized Team Cognition

Although we have claimed the essentiality for examining artifacts as a part of team cognition, we so far, have yet to elaborate on what we mean when we refer to artifacts and the evidence for their value to team cognition. Therefore, in this section, we provide a foundation for conceptualizing artifacts, and the varied ways in which they’ve been viewed, as a form of external cognition. In addition, we also review various technologies that have been developed to support teams in a number of domains that are characteristic of artifacts that facilitate external cognition. This section illustrates how evidence for this area of inquiry has been independently developing in a variety of fields that do not always influence each other and show how to leverage these developments to integrate ideas on external cognition with team research in the organizational sciences.

The notion of the *cognitive artifact* emerged in studies of design and human–computer interaction and was characterized by Norman (1991, p. 17) as an “artificial device designed to maintain, display, or operate upon information in order to serve a representational function.” Importantly, there is a long history in the social sciences of conceptualizing artifacts as a means for supporting human capabilities. As noted by Norman (1991), a number of theoretical positions emerging in the 20th century, such as “activity theory” or “situated action,” focused on the role of the natural and artificial environment in enhancing human abilities (for a review, see Fiore, 2013). Even early work on information processing theory discussed how representations as external symbol systems foster complex cognitive processes (Larkin and Simon, 1987). Here, diagrammatic representations were said to group related information and minimize problem space search and also support perceptual inferences about processes. These and related features of externalization were argued to make cognitive processes more computationally efficient. This early thinking influenced research where the focus was on cognition in work contexts as well as in learning and training research. In these varied settings, the concept of an artifact has fallen under a number of labels, but all related in the sense that they are a form of external cognition. We next briefly review these in turn.

#### Artifacts in Distributed Cognition

An influential early theory with representational information at its core is Hutchins (1995a,b) theorizing on distributed cognition. The primary argument is that cognitive processes are not only internal, but are also spread across task and environmental artifacts, as well as team members. Heavily based on information processing theory, cognitive processes were said to act on these representations via computation of some form to transform understanding. Cognitive artifacts were defined as “physical objects made by humans for the purpose of aiding, enhancing, or improving cognition” (Hutchins, 1999, p. 126). With this, the focus was on the interaction of distributed structures in a broader cognitive system. As one example, Hutchins used cockpit technology (e.g., attitude indicator) and the aviation crew, to describe a distributed cognitive system. In these specific settings, some have even discussed the idea that automation technology be construed of as a teammate (Hoeft et al., 2006). This expanded the boundaries of how cognition can be analyzed – with distribution encompassing processes across time, as well as across the team, and internal and external cognitive structures in humans, and their supporting technology.

This was further detailed in the context of human–computer interaction research, where an ethnographic approach was used to study the use of digital artifacts that trace histories of interaction (Hollan et al., 2000). Here, distributed cognition was examining the interplay of internalized and externalized cognition “involving coordination at many different time scales between internal resources—memory, attention, executive function—and external resources—the objects, artifacts, and at-hand materials constantly surrounding us” (Hollan et al., 2000, p. 177).

Research in healthcare teams also examined the role of cognitive artifacts in supporting coordination across team members (Nemeth et al., 2004, 2006; Rambusch et al., 2004). In this context, it was shown how technologies assisted teams in the form of externalizations such as team schedules, lists, display boards, and patient records. Similarly, in the context of emergency rooms, externalization of cognition, through the use of whiteboards, was shown to support coordinating responsibilities and resources. In short, artifacts in the form of visual representations, act as aids to memory and provide information directly perceivable by members of the team to facilitate collaboration. These forms of external cognition helped teams maintain a shared overview of the total team activity distributed across time, location, and across different technologies (Nemeth et al., 2004). External cognition has also been shown to help teams dynamically make decisions and identify potential problems that might arise in their task (Xiao et al., 2007). This and related work has been used to help system designers understand how artifacts could be transitioned to digitally based forms to create a more resilient system.

Collectively, this works shows how artifacts support activities like team planning by mediating collective work and the management of resources (Nemeth et al., 2004). They further elucidate how this can vary as a function of *who* was using a given artifact and *where* (Rambusch et al., 2004). Taken together, this research provides a foundation for seeing teams, their technology, and the resultant externalizations, as a distributed cognitive system (cf. Hutchins, 1995b).

#### Boundary Objects in Organizational Research and Computer Supported Cooperative Work (CSCW)

In the organizational sciences, the concept of materiality and sociomaterialy are often used to capture how some artifact, loosely defined, influences, and is influenced by, work processes. This body of research examines organizational functions at a broad level (e.g., finance), and how technology relates to that (e.g., spreadsheet software). And much work has gone in discussion and debate on what is meant by materiality and related terms (Leonardi, 2010, 2012). Theoreticians have debated how to conceptualize this idea and its relation to the material part of organizations. Here they argue that, “whereas materiality might be a property of a technology, sociomateriality represents that enactment of a particular set of activities that meld materiality with institutions, norms, discourses, and all other phenomena we typically define as ‘social”’ (Leonardi, 2012, p. 34).

Relevant to this paper, reviews in the organizational sciences note that most studies in areas relevant to cognition (e.g., decision-making, strategic thinking), *have not* considered how technology influences these complex processes (Orlikowski and Scott, 2008). Similarly, some have argued that organizational research needs to better integrate ideas about how information technology and the materiality it affords, is related to the functions and processes of organizations (Leonardi and Barley, 2008). This work shows the far reaching recognition that externalizations provide a powerful means of connecting people.

Despite the conceptual connection of such ideas to the notion of artifacts, socio-materiality operates at a level above teamwork. That is, it transcends work in teams and represents objects that connect, not necessarily individuals within a team, but groups of people within an organization, and even entire communities of practice. As such, this body of research has not had an influence on, let alone been integrated with, team cognition. But fields that focus more on technology and its relation to team functions [e.g., Information Systems, Computer Supported Cooperative Work (CSCW)], come close to addressing this gap through the development of the concept of *boundary objects*. As such, we next turn to a description of research in boundary objects to set the stage for how this can be related to team cognition.

Within the field of CSCW, a significant amount of research has been on the development and use of what were termed material resources (see Blomberg and Karasti, 2013, for a review). These ranged from artifacts as simple as paper documents to computer displays and whiteboards and maps. Early work in this area showed how these help collaborators align their activities by drawing attention to coordination needs (Suchman and Trigg, 1991; Heath and Luff, 1992). Some have used the generic label of “shared representation” to capture this concept. These are external representations that arise in collaborations and can vary in meaning and relevance depending on context in which they are used (see de Vries and Masclet, 2013). Out of such work arose a particular form of sociomaterialy, known as boundary objects. Originating in research on scientific work, these were described as practical artifacts that mediate interaction across diverse groups and communities of practice with varying expertise and perspectives (Carlile, 2002, 2004; Yakura, 2002; Hecker, 2012). These “tangible” artifacts were shown to act as a bridge from which communication and coordination occur, thus facilitating not only the transfer of knowledge from an individual to a team level, but also the maintenance of shared representations (Yakura, 2002; Nicolini et al., 2012; Stigliani and Ravasi, 2012).

This concept has had an influence in a number of domains as it has been adopted by researchers in the organizational and information sciences (see Lee, 2007 for a review). Early research on boundary objects suggests that they foster cooperation between diverse communities of stakeholders through creation of a shared identity (Star and Griesemer, 1989; Star, 2010). Additionally, boundary objects were seen as a means of both knowledge transfer, and a method for translating meaning across an organization utilizing shared information systems (Carlile, 2004). Some have looked at this in the context of, not just the development of information systems, but also their implementation (Doolin and McLeod, 2012). Here, it was argued that communities of practice needed to develop competencies *about* boundary objects so that those working in these settings could make them useful (Levina and Vaast, 2005).

From this, CSCW researchers described the development of “common information spaces” that help make explicit “the interrelationships between information, workers, and artifacts… [and] involve the joint interpretation of and the meaning attributed to these artifacts and representations” (Blomberg and Karasti, 2013, p. 382). And out of this came the notion of “coordinative artifacts” that were seen as essential to collaboration in complex cognitive work (Schmidt and Wagner, 2004; Lee, 2007). These were argued to reduce the amount of articulation of what needed to be done by specifying division of labor as well as sequencing/ordering of activities (Bardram and Bossen, 2005). Along with this was the need for active negotiation about a boundary object in order to develop shared understanding (Lee, 2007). These served different roles dependent upon the task needs – ranging from the simple, such as including ideas or compiling ideas (e.g., tables), to the more complex, such as structuring ideas (e.g., concept maps). These were said to serve either a syntactic function to help collaborators transfer knowledge via a common vocabulary, or a semantic function that helps identify differences in knowledge to create shared knowledge (Carlile, 2004). In brief, these allow collaborators to “record, organize, explore and share ideas; introduce concepts and techniques; create alliances; create a venue for the exchange of information; augment brokering activities; and create shared understanding about specific problems” (Jirotka et al., 2013, p. 668).

Research has also examined how these forms of external cognition help orient team members in complex decision making tasks. In a study of argument representation and patient diagnosis, research on medical decision making used an interactive whiteboard and studied how it enabled team members to represent perspectives about data (symptoms and vital signs) as well as about solutions in the form of diagnoses (Lu et al., 2010). In problem solving research, network visualization tools have been examined as a means of promoting communications in distributed teams (Balakrishnan et al., 2008). Visualization tools support both individual and team problem solving by providing shared access to data in an externalized form (representations illustrating data as nodes). Further, these tools foster an increase in information sharing among team members that helps them better “connect the dots” and develop a shared understanding of the problem.

More recent research has explicated a catalog of action patterns and a variety of complex cognitive activities that can be utilized for technological visual representation tools to support teams (Sedig and Parsons, 2013). Studies on display design have shown how variations in externalizations influence complex collaboration. For example, a translucent interface meant to assist sensemaking, fostered collaboration by supporting the sharing of insights and preventing narrowing of focus (Goyal and Fussell, 2016). This led to collaborators identifying more problem solving clues as well as finding a target in a criminal investigation task.

Technical domains such as architecture have also been studied to understand how externalizations foster technical work in the design process. Here, research has found that architects varied in their use of high- vs. low-resolution drawings dependent upon both task needs and the people with whom they were communicating (Retelny and Hinds, 2016). For example, architects were found to use these for both conceptual work with clients and for technical work with design teammates. In the former case, high-resolution representations supported the development of mutual understanding of the project’s “design intent” as well as helped with collaborative decision making. In the case of the latter, low-resolution images would be used to provide and elicit feedback as well as resolve misinterpretations or ambiguities.

This concept has also been used in the context of scientific collaboration to show how boundary objects support interdisciplinary research. Science teams are found to create visual models and co-construct diagrams while engaged in collaborative processes (Pennington, 2010). The line of work has also integrated the idea of boundary objects with model-based reasoning to describe how scientists from different disciplines create boundary negotiating objects that support development of shared understanding (Pennington, 2011a,b). In line with early theorizing on shared problem model development (Fiore and Schooler, 2004), Pennington et al. (2016) have shown how external representations provide a firm foundation on which collaborators are able to create mutual understanding of complex problems.

In sum, boundary objects can be characterized as extern alizations of cognition and may take the form of drawings, charts, graphs, prototypes, or models generated by team members, as well as tools used for project management, such as timelines and Gantt charts, or schedules and tables (see Ewenstein and Whyte, 2009). This work fits with research noting that information technology can be construed of as a form of transactive memory system (Lewis and Herndon, 2011). In the context of collaboration, the technology acts as an external memory system that is relied upon to support team processes. We suggest that boundary objects, as a technology-based form of transactive memory, can be viewed as serving the explicit purpose of facilitating coordination and collaboration between the functional or disciplinary boundaries of team members. This is particularly important for team effectiveness in that this is where common ground is not frequently held (cf. Bruns, 2013).

#### Representations in Training and Learning Research

While the aforementioned research looked at externalized cognition in support of teamwork in various complex work contexts, evidence for it also comes from research on training and learning. External representations in the form of “information boards” were used in a training study of knowledge building for a collaborative planning task (Rentsch et al., 2010). Information boards supported the creation of artifacts in the form of posts and allowed team members to organize and visually manipulate these posts. Further, it allowed them to focus shared attention on particular facets of knowledge when appropriate. Training with these artifacts supported team member transfer of knowledge and knowledge congruence, leading to overall improvements in team performance (Rentsch et al., 2010). In related research, Rentsch et al. (2014) studied training in the use of knowledge objects in collaborative problem solving. These were artifacts designed to foster schema-enriched communication in the team chats. This fostered the sharing of unique information and the transfer and congruence of knowledge across the team, leading to superior solutions.

Technology supported learning research has also been studying the externalization of cognition during collaboration. Here, visualization tools are used to externalize cognition in the form of representational artifacts such as diagrams, maps, or sketches, that help team members better understand task elements and their relations. For example, early research examined how computer support tools allow team members to jointly construct representations (Roschelle and Teasley, 1995). They found that these facilitated the definition of the problem space and the explication of executable problem solving plans. Other research has shown how computer-based visualization tools, such as matrices and graphs, are effective in helping teams learn about the connections between data, hypotheses, and evidential relationships (Suthers and Hundhausen, 2001). In a discussion of group cognition in the context of technology and learning, Stahl (2006) lays out a framework for understanding the interaction between individual and collective cognition, negotiation of meaning and understanding and how technology supports knowledge building.

Others have focused on developing technologies that help structure arguments to support learning. For example, representational tools that help information checking was found to be constructive (Kanselaar et al., 2002). Further, collaborative learning teams were shown to need help coordinating their communications as well as help in being kept on track with regard to their argumentation processes because they would often lose their thematic focus (for an early review of these tools see Kanselaar et al., 2002). Such approaches can also more specifically help teachers understand and support collaborative cognition in their classes. In an online computer science course, visualization tools were used to represent student processes and were found to help the teacher develop a better awareness of the class’ performance and for students to develop self-reflection skills (Govaerts et al., 2010).

In sum, technologies that support external cognition in a learning context (e.g., ‘mindtools,’ visualizations, concept maps), are argued to augment knowledge acquisition by helping learners more easily represent their knowledge. With these externalizations, learners develop a shared representation that can help them transform data and information into knowledge around the content to be learned. This transformation takes place through interpretive activities, such as critical thinking or manipulative visualization, around the representations (Kirschner and Erkens, 2006).

#### Summary

In sum, this review was meant to provide evidence for the external cognition perspective as it has emerged somewhat independently in a variety of domains. Although referred to with differing terms, thematically similar across these studies is the role of cognitive artifacts and various technologies supporting a variety of teamwork processes in numerous fields (see **Table 1**). Stated simply, the items reviewed above are exemplars of, and evidence for, the concept of external cognition. This is the case primarily in the sense that the required cognitive activity is distributed among members of a team and their task elements, in which artifacts serve to coordinate between internal and external structures over some duration of time (cf., Hutchins, 1995a; Hollnagel, 2002; Fiore et al., 2010b; Cooke et al., 2013; Stanton, 2016).

**Table 1:** Representative descriptions and forms of artifacts arising in varied research literatures.

Area of research	General definition	Example forms	Reference
*Cognitive Artifacts in Distributed Cognition*	Cognitive artifacts are conceptualized as something constructed by humans as an aid to enhance or improve cognitive processes	Schedules, lists, display boards, patient records, digital traces, navigation technology	Hutchins, 1995a,b; Hutchins, 1999; Hollan et al., 2000; Nemeth et al., 2004, 2006; Rambusch et al., 2004; Zhang and Patel, 2006; Xiao et al., 2007
*Boundary Objects in Organizational Research and Computer Supported Cooperative Work>*	Boundary objects are practical artifacts that mediate interaction and shared knowledge across diverse groups and communities of practice with varying expertise and perspectives and act as a bridge for communication and coordination	Paper documents, technical drawings, whiteboards, maps, tables, computer displays, software algorithms; network diagrams, information spaces, coordinative artifacts	Star and Griesemer, 1989; Suchman and Trigg, 1991; Heath and Luff, 1992; Carlile, 2002, 2004; Yakura, 2002; Fiore and Schooler, 2004; Schmidt and Wagner, 2004; Bardram and Bossen, 2005; Levina and Vaast, 2005; Lee, 2007; Balakrishnan et al., 2008; Leonardi and Barley, 2008; Orlikowski and Scott, 2008; Ewenstein and Whyte, 2009; Leonardi, 2010, 2012; Lu et al., 2010; Star, 2010; Pennington, 2011a,b; Doolin and McLeod, 2012; Hecker, 2012; Nicolini et al., 2012; Stigliani and Ravasi, 2012; Bruns, 2013; Blomberg and Karasti, 2013; Jirotka et al., 2013; Goyal and Fussell, 2016; Retelny and Hinds, 2016
*Representations and Visualization in Training and Learning Research*	Representations are forms of visualization that focus shared attention on learning elements and support development of arguments or richer knowledge structures by showing relationships across elements of the to-be-learned content	Information boards; diagrams, maps, sketches; matrices, graphs, mindtools	Roschelle and Teasley, 1995; Zhang, 1998; Suthers and Hundhausen, 2001; Kanselaar et al., 2002; Zhang and Wang, 2005; Kirschner and Erkens, 2006; Stahl, 2006; Govaerts et al., 2010; Rentsch et al., 2010, 2014

All this is to say that artifacts play an essential role in teamwork, and as such, leveraging the notion of external cognition to improve team process requires integrating concepts from the organizational sciences on the study of teams with relevant ideas from cognitive science. As a theoretical mechanism, then, the construct of external cognition can be conceptualized and measured as something supporting inter-related team functions (Salomon, 1993; Zhang and Norman, 1994; Zhang, 1997; Zhang and Wang, 2005; Zhang and Patel, 2006). Despite the evidence, this body of research has yet to be integrated with important concepts from organizational research on different elements of teamwork.

Toward this end, we next draw from these varied literatures, and link the findings described above to the distinction found in organizational research between “teamwork” and “taskwork.” With this, we show how they can help parse different aspects of team cognition, particularly the role played by artifacts in the team environment. With a clearer description of artifacts and technological tools, coupled with concepts from team research, external cognition can be more fully integrated with team theory to understand how technology can be conceptualized and measured *as* a teammate. From this, we inform new avenues of research for team cognition to examine the ways artifacts support processes and enhance performance in the context of hybrid human technology teams.

## Integrating the Organizational and Cognitive Sciences in the Study of Team Cognition

Research in teams and team training has provided a solid foundation on which to understand and improve team process and outcomes. However, as noted, external cognition, and the role that technology plays in facilitating team processes, has yet to be fully integrated in much organizational research. In this section, we attempt to partially redress this gap by describing a framework from team theory that can be used to conceptualize and measure external team cognition, which, in turn, could inform the design of technology meant to support team performance. By adopting and adapting concepts, we contribute to team cognition research by helping to more precisely determine the role artifacts play in team process and how technologies mediate the creation and use of such artifacts.

### Teamwork and Taskwork in Team Cognition

The distinction between teamwork and taskwork in the organizational sciences has been a useful heuristic for conceptualizing collaboration in teams (e.g., Cannon-Bowers et al., 1995; Mathieu et al., 2000). *Teamwork* is characterized as the types of behavior essential for working together with team members including designated roles and responsibilities, interdependencies of team members, and communication patterns. *Taskwork* is characterized as the necessary functions required for meeting objectives such as the operating procedures for equipment, strategies for achieving goals, and the relationships between sub-components of a task (Mathieu et al., 2000).

Adopting this important conceptual distinction helps us to categorize the forms of external cognition detailed previously. Specifically, we can more precisely articulate clear distinctions regarding the role of externalized cognition in supporting either teamwork or taskwork. On the one hand, artifacts can support *teamwork* by providing novel and more articulated ways for understanding the workflow of the team, conveying dynamic plans, and overall, clearly displaying how the work is done (e.g., Nemeth et al., 2004; Ewenstein and Whyte, 2009) as well as facilitate communication, coordination, and shared representations across multi-disciplinary teams (Yakura, 2002). On the other hand, artifacts can support taskwork by providing novel tools for analyzing data (Suthers and Hundhausen, 2001), interpreting information (Balakrishnan et al., 2008), solving problems (Roschelle and Teasley, 1995), and making decisions (Lu et al., 2010). In short, with this distinction of teamwork and taskwork, we can illustrate *what*, specifically, the external cognition is supporting. We can take this a step further with the distinction between generic and specific competencies of teamwork and taskwork to add even greater precision for the study of team cognition.

#### Generic and Specific Competencies in Teamwork and Taskwork

An additional framework from the organizational sciences that can be used to guide our understanding and measurement of external team cognition is one that explicates the team and task *competencies* necessary for successful team performance (Cannon-Bowers et al., 1995). This framework outlines how certain competencies are required in virtually all team situations, whereas others are specific to certain teams (Bowers et al., 2000). In the former, all team members need what are referred to as *team-generic competencies* regardless of the task context or the organizational setting (e.g., communication skills). In the latter, some competencies are considered to be team-specific, as they are argued to apply in only particular situations. These *team-specific competencies* are more directly related to individual teams and include knowledge of roles within the team and the abilities held by team members (Bowers et al., 2000). Relatedly, task characteristics can also be thought of along these dimensions; namely, task-generic and task-specific competencies. Whereas *task-generic competencies* are those that are necessary across task situations (e.g., exchanging information and planning), *task-specific competencies* could include understanding the goals of a certain task or the appropriate methods for accomplishing that task.

#### Integrating Teamwork/Taskwork Theory with External Cognition

This synthesis of the teamwork and taskwork concepts, combined with the notion of generic and specific team and task competencies, provides an important conceptual grounding for understanding and measuring external team cognition. By more precisely describing how artifacts and the technology used to manage them can support teams, we provide guidance on how to study team cognition within sociotechnical systems. We expect that this can be used to produce a more detailed understanding of the team processes supported by technological artifacts as they relate to the needs of specific teams as well as those that are more generic for all teams (see **Table 2**). In turn, this allows for more fine-grained theoretical specification and testing within team cognition research. Based upon this integration, and to lay the groundwork for theory development, we next provide a set of propositions for team cognition research that takes into account the role of artifacts. These are devised to unite these perspectives so as to better study teams in complex settings through a more detailed examination of the types of external cognition that a given technology can support.

**Table 2:** Team and task competencies propositions for externalized cognition.

		Relation to the task	
		*Specific*	*Generic*
**Relation to the team**	***Specific***	P1. Context Driven	P2. Team Contingent
	***Generic***	P3. Task Contingent	P4. Transportable

• **Proposition 1 – *Context driven technology*.** We propose that the effectiveness of context driven technologies that produce externalizations are dependent upon the degree to which they are specific to both the team and the task. These should support, for example, an understanding of the team’s goals and their resources. Additionally, these should help with managing whether or not the team is monitoring progress and meeting objectives.
• **Proposition 2 – *Team contingent technology*.** We propose that the effectiveness of team contingent technologies for external cognition are dependent upon the degree to which they are specific to a team, but generic to a task. These should more generally enable a team by supporting team processes like conflict resolution. However, these would do so while managing teammate specific characteristics. For example, such a system might be able to track the degree to which team members have expertise in a particular topic and leverage that knowledge to inform and guide resolutions to disagreements.
• **Proposition 3 – *Task contingent technology*.** We propose that the effectiveness of task contingent technologies supporting externalization are dependent upon the degree to which they are specific to the task but generic as to the team. These should support the completion of particular procedures that are relevant to a large number of teams. These could support analyzing particular forms of data teams might need to make decisions. For example, in healthcare teams, these would support review of patient records, or collaborative evaluation of tests like x-rays, etc. in support of diagnosis; but any healthcare team could use them.
• **Proposition 4 – *Transportable technology*.** We propose that the effectiveness of transportable technologies for externalization are dependent upon the degree to which they are generic to a team and to a task and could support any form of team/task. These could include, for example, scheduling systems for completion of tasks or these could be a means of supporting communication processes across distributed teams. The point is that it is a more general form of technological aid that could have a broader impact on team process and performance without the need to be tailored to any particular context.

By drawing from established theory in team research, this section was meant to provide greater specificity to the team and task functions external cognition is supporting, that is, a description of *what* externalizations are supporting. In this way, we are able to better specify the role of artifacts in team process. As such, the teamwork/taskwork framework and the associated generic/specific competencies, help to conceptualize how technology can sometimes be seen as a teammate in the context of hybrid human-technology teams.

### Oﬄoading and Scaffolding in Team Cognition

Whereas the prior section considered *what* technology and associated artifacts might support as part of team cognition, we next discuss *how* they could support cognitive processes of a team. We integrate the teamwork and taskwork dimensions with theory from the cognitive sciences to provide potential explanatory mechanisms for how artifacts developed and/or used by teams support process and performance. Specifically, the externalized view of cognition provides two constructs that help us better understand important aspects of team process and performance: *oﬄoading* and *scaffolding* (Clark, 2008).

*Oﬄoading* is generally the act of using the environment as a semi-permanent archive for information that can be readily available and accessed when needed, but it also used to mitigate encoding and short-term memory demands (Wilson, 2002). As such, oﬄoading primarily serves the purpose of a memory aid that can free up cognitive resources that can then be allocated toward other team processes. In this sense, it replaces what was previously an internal form of cognitive processing such as holding an item in working memory or retrieving something from long-term memory. Often seen as an evolutionary adaptation, and by some, the center of human intelligence (Dennett, 1996), oﬄoading fits with our points about context and cognition reviewed earlier in that it allows for efficient utilization of the environment to reduce the complexity of memory-intensive problems (Parsell, 2006).

*Scaffolding* takes the form of externalizations of cognition that directly support team-level processes by helping to mediate and support the interaction between individual and team-level cognitive activity. Scaffolding, in this sense, supports social interaction broadly (Baron, 1991; Krueger, 2011), as well as the analysis, discussion, debate of items relevant to the team’s task, and the development of the teams shared understanding (e.g., Fiore and Schooler, 2004). Specifically, technological scaffolds can help teams externalize and share knowledge by allowing for the representation and discussion of information and ideas, provide storage and access to team-level information allowing for more informed comparisons and evaluations, and act as a means for social-cognitive interaction that facilitates conversation, communication, and collaboration (McLoughlin and Luca, 2002). Further, given the virtual nature of many modern day teams, and how varied forms of technology connect such teams, scaffolding is essential for effective coordination when teams work across time and space (Fiore et al., 2003; Miles and Hollenbeck, 2013).

Indeed, it is our capability to engage in oﬄoading and scaffolding that is what some argue to be a distinctly human trait. Further, these can be seen as a primary means through which we have made great advances in civilization because of the innovations in thinking they afford. Specifically, “our habit of oﬄoading as much as possible of our cognitive tasks into the environment itself—extruding our mind (that is our mental projects and activities) into the surrounding world, where a host of peripheral devices we construct can store, process, and re-represent our meanings, streamlining, enhancing, and protecting the processes of transformation that are our thinking” (Dennett, 1996, pp. 134–135), has significantly expanded our cognitive capabilities beyond the limitations of our biology.

#### Integrating Oﬄoading and Scaffolding for External Team Cognition

Adding oﬄoading and scaffolding to the team cognition literature has both theoretical and practical benefit. From the theoretical standpoint, these concepts provide a means to better understand the form of team cognition as it is emerging in complex work settings. As such, it helps us to better conceptualize *how* artifacts and technologies are enabling differing kinds of team process and/or performance outcomes (e.g., Rosen, 2010; Wiese et al., 2011). From the practical standpoint, the adoption and adaptation of these constructs from the cognitive sciences will provide greater precision in, and mechanisms for, measuring external team cognition.

To guide examination of the relation between team cognition and technological artifacts, we provide the following research questions for assessing external team cognition as a means of oﬄoading or as scaffolding. These are provided to show how theorizing from cognitive science can help lay the groundwork for research on technological supports designed to improve process and performance of teams as sociotechnical systems (see **Table 3**).

**Table 3:** Framework for guiding research on external cognition.

Focus of support	Role of external cognition	
	*Offoading*	*Scaffolding*
***Taskwork***	R.Q. 1.1 and 1.2	R.Q. 3.1 and 3.2
***Teamwork***	R.Q. 2.1 and 2.2	R.Q. 4.1 and 4.2

1. **Are technologies providing externalizations supporting *taskwork* through oﬄoading?**
  • *Research Question 1.1.* Are technologies devised to support memory based elements of the task (e.g., storage of operating procedures) being relied upon by team members to meet their objectives (e.g., accessing a manual for trouble shooting)?
  • *Research Question 1.2.* Are technologies devised as repositories for task relevant data (e.g., bulleted lists of relevant information for later use) being used by team members to meet task needs?
2. **Are technologies providing externalizations supporting *teamwork* through oﬄoading?**
  • *Research Question 2.1.* Are technologies devised to support understanding of team process (e.g., graphical representation of workflows across members) being used by the team to meet their objectives?
  • *Research Question 2.2.* Are technologies devised in support of communication (e.g., mapping who knows what and related role interdependencies) being relied upon as a guide for team related knowledge?
3. **Are technologies providing externalizations supporting *taskwork* through scaffolding?**
  • *Research Question 3.1.* Are technologies devised to support task-relevant activities (e.g., dynamically updated visualizations to guide information interpretation) being used by team members as they interact to work toward goals?
  • *Research Question 3.2.* Are technologies devised to support understanding of task-related elements (e.g., visualizations helping to illustrate relations between data), being utilized as they collaborate to meet objectives?
4. **Are technologies providing externalizations supporting *teamwork* through scaffolding?**
  • *Research Question 4.1.* Are technologies devised to support interaction processes (e.g., helping to represent ideas around arguments to foster constructive conflict) being relied upon in service of their teamwork?
  • *Research Question 4.2.* Are technologies devised to support team outcomes (e.g., helping teams evaluate solution alternatives to reach consensus) being used to meet performance outcomes?

In sum, we have provided this representative set of questions in such a way that researchers can see how to integrate the concepts of oﬄoading and scaffolding with their own theorizing on team cognition. By framing these within the context of teamwork and taskwork as well as oﬄoading and scaffolding, we offer theoretical concepts that, themselves, can augment existing theory. In this way, sociotechnical systems research can make significant strides in understanding and explaining the ways in which artifacts, and the technologies supporting their development and use, can be construed of as part of a larger system that is, essentially, a team-technology hybrid.

## Discussion

Our goal with this paper was twofold. First, we set out to provide an overview of the externalized view of cognition in the context of team process and performance. We add to theory that views cognition within individuals and within teams as something spanning team members and their technology (Hutchins, 1995a; Hollnagel, 2002; Stanton et al., 2006; Fiore et al., 2010b; Cooke et al., 2013). Second, we lay the groundwork for future research to consider and measure this type of cognition as it occurs across individuals, team members, technologies, and artifacts. We expect that adopting this approach will lead to an enriched perspective of team cognition theory that will augment many lines of research and measurement methods. We have provided preliminary progress toward this by integrating ideas on external cognition with insights from varying disciplines that have, so far, shown little integration. Specifically, on the one hand, we have drawn from team theory in the organizational sciences to articulate how research can examine *what* and *where* technology and artifacts support team process and performance; that is, teamwork and taskwork. On the other hand, we have drawn from the cognitive sciences to articulate the ways research can examine *how* technology and artifacts support team process and performance; that is, oﬄoading and scaffolding.

Through this integration, we are able to connect related concepts from across a disparate set of disciplines. Foundational to this was the need to illustrate how team cognition researchers could leverage ideas emerging from fields ranging from cognitive engineering, to computer supported collaborative work, to the organizational and cognitive sciences. Specifically, by blending theory from research on teamwork (Cannon-Bowers et al., 1995), with concepts from the organizational sciences (e.g., Carlile, 2004; Hecker, 2012), the cognitive sciences (e.g., Zhang and Norman, 1994; Clark, 2001a), and cognitive engineering (Hollnagel, 2002; Fiore et al., 2010b), we provide a framework for understanding and measuring how team process and performance is altered through the use of artifacts and technology. This was framed within recent theorizing on MiTs, which takes into account the role of artifacts in both internalized and externalized cognition (Fiore et al., 2008, 2010b,c), as well as theorizing on extended cognition (Clark and Chalmers, 1998; Clark, 2001a).

Note that this approach is different from others who have construed of teams *as* technology (Wallace and Hinsz, 2010). In that line of theorizing, teams are, themselves, viewed as a form of technology that is used to transform internalized cognitive resources into team solutions. Likewise, while we share similar views on team cognition with ITC theory (Cooke et al., 2013; Cooke, 2015), and DSA (e.g., Stanton et al., 2006), our focus and contribution here is distinct. That is, we elaborate upon, and extend, such efforts by making explicit the systemic relationship between team cognitive processes and the types of technological artifacts that facilitate both the externalization of knowledge and effective team performance. Our approach adds to such thinking in that we broaden what role technology potentially plays in team cognition and, indeed, should be seen as a fundamental element of the team.

As such, our efforts support recent calls by those in the organizational sciences to develop a richer understanding of the modern workplace and the complexities inherent given the role of technology in business processes (Juillerat, 2010; Bell and Kozlowski, 2012; Stigliani and Ravasi, 2012; Kozlowski et al., 2015). Further, this framework supports recent work in the study of scientific collaboration and the interaction of people and technology in support of innovation (e.g., Fiore, 2008; Asencio et al., 2012; Cummings et al., 2013).

An additional implication of the integration we have provided is that it can help to broaden current understandings of team cognition and how it is conceptualized and measured. Meta-analytic studies have shown how aspects of team cognition (e.g., shared mental models) are predictive of team process (DeChurch and Mesmer-Magnus, 2010b; Turner et al., 2014) and furthered our understanding of how compositional and compilational variables relate to team process and performance (DeChurch and Mesmer-Magnus, 2010a). We have provided a more precise framework for understanding the form and role of technological artifacts in team cognition. This broadens our conceptualization of what can be part of a shared mental model and/or what role technology plays in transactive memory systems (Austin, 2003; Lewis, 2003; Zhang et al., 2007; Huber and Lewis, 2010; Lewis and Herndon, 2011; Tollefsen et al., 2013), and cross-disciplinary coordination and collaboration (e.g., Susi et al., 2003; Gittell and Weiss, 2004; Gittell, 2006; Rico et al., 2008; Okhuysen and Bechky, 2009). These ideas can also fit within new methods for measuring teams, such as social network analysis (e.g., Leenders et al., 2016). For example, it is possible to see how artifacts utilized by teams can be viewed as nodes in a network that are part of collaboration. Thus, our accounting provides guidance on future efforts to advance the science of teams in complex sociotechnical settings by detailing additional factors for studying mediation and moderation in meta-analytic work on team cognition and suggests ways to inform the design of new tools for enhancing both team process and performance.

Viewed more broadly, our focus can be seen as an argument that technology, broadly construed, needs to be taken more seriously as a member of a team. The conceptual frameworks we put forth in this regard, are timely in that technology is going to play an increasing role in teams. We are now seeing semi-autonomous robots as members of teams in complex and high-stakes environments. For example, the military is making use of them in settings such as explosive detonation whereas, in civilian settings, robots are playing a significant role in areas such as search and rescue. Furthermore, organizations will soon be confronted with the reality of intelligent technology, in various forms, in the workplace. Whether this be in the form of cognitive computing and artificially intelligent support systems (e.g., stock trading; medical diagnosis), or embodied robots interacting on the factory floor, autonomous systems will need to be studied by organizational scientists. We provide a foundation on which to more fully examine how these systems will function as a member of a team and provide conceptual grounding for the next evolution of team research.

In this context, our integration provides a foundation for the next phase of team cognition research, a phase that will increasingly be studying hybrid human-technology teams (e.g., Wiltshire and Fiore, 2014). We provide a scaffold for understanding, not just how humans draw from, and rely on, technology in the context of teams. We additionally provide a foundation for the coming infusion of new technologies (e.g., cognitive computing, robotics) in organizational settings. The prevalence of artificial intelligence in computing systems (e.g., decision support), and machine production (e.g., industrial robotics), will only become more commonplace in the workplace. Because of this, researchers in team cognition need to have the conceptual scaffolds that well help link these technology developments to their theorizing and increase our understanding of team effectiveness.

Further, the current framework calls for, and contributes to, new forms of interdisciplinary research in the study of teams by helping to develop additional ways to conceptualize and measure team cognition. As the sophistication of technology continues to advance, and as humans continue to integrate these advances in their lives, we must, ourselves, become more sophisticated in how we study these phenomena. As Clark (2001a) articulated so well, collaboration between humans and technology should be viewed as a *continuous reciprocal causation*; specifically:

Much of what matters about human intelligence is hidden not in the brain, nor in the technology, but in the complex and iterated interactions and collaborations between the two.… The study of these interaction spaces is not easy, and depends both on new multidisciplinary alliances and new forms of modeling and analysis. The pay-off, however, could be spectacular: nothing less than a new kind of cognitive collaborative collaboration involving neuroscience, physiology, and social, cultural, and technological studies (Clark, 2001a, p. 154).

We further this view to suggest the need for an externalized view of cognition as it relates to teams and their associated teamwork and taskwork. In doing so, external cognition provides a means for enriching study of the interdependencies across both individuals and teams and their use of artifacts and technologies such that the team competencies required for effective performance can be more fully examined. Further, when these complementary distinctions are integrated, this can better inform our understanding of the role of technological support systems in team cognition.

## Conclusion

For researchers in sociotechnical systems, we emphasize that an interdisciplinary collaboration between the cognitive, organizational, and computational sciences is needed. Such research would, not only be aimed at understanding and enhancing team process and performance, but would also serve the design and delivery of approaches that better support teams in many of society’s current, and future, complex socio-technical systems.

## Author Contributions

All authors listed, have made substantial, direct and intellectual contribution to the work, and approved it for publication.

## Conflict of Interest Statement

The authors declare that the research was conducted in the absence of any commercial or financial relationships that could be construed as a potential conflict of interest.
